# Cooperation in bottlenose dolphins: bidirectional coordination in a rope-pulling task

**DOI:** 10.7717/peerj.7826

**Published:** 2019-10-02

**Authors:** Chisato Yamamoto, Nobuyuki Kashiwagi, Mika Otsuka, Mai Sakai, Masaki Tomonaga

**Affiliations:** 1 Primate Research Institute, Kyoto University, Inuyama, Japan; 2 Kagoshima City Aquarium, Kagoshima, Japan; 3 Faculty of Agriculture, Kindai University, Nara, Japan

**Keywords:** Cooperation, Bottlenose dolphins, Problem solving

## Abstract

In comparison with terrestrial animals, such as primates, there is limited empirical evidence for cooperative behavior in marine mammals under experimental conditions. In this study, we used a cooperative rope-pulling task to investigate how bottlenose dolphins (*Tursiops truncatus*) coordinate their behavior with a partner. Dolphins successfully learned and were able to perform the task, even when one subject started after the other. In the no-delay condition (i.e., both subjects sent at the same time), one pair of dolphins showed coordinated behaviors. When pairs were successful in solving the task in the delay condition (i.e., one individual sent later than the other), the initiators (i.e., first individual sent) were likely to wait for the follower to arrive, and the follower was likely to swim faster when the initiator did not wait and started pulling the rope alone. These coordinated behaviors might help resolve the given cooperative task. Our results suggest that bottlenose dolphins learn to coordinate their behaviors via trial and error and recognize the necessity of performing simultaneous actions with a partner to successfully accomplish cooperative tasks. In addition, both partners showed behavioral changes over many trials of no-delay and delay conditions, suggesting that bidirectional coordination occurred in the cooperative task.

## Introduction

There have been many studies on various aspects of social intelligence in primates, other terrestrial mammals, and birds (Byrne & Whiten, 1988; De Waal & Tyack, 2003; Seed, Emery & Clayton, 2009). Cooperation is one of the most important social cognitive abilities for social species (Byrne & Whiten, 1988; De Waal & Tyack, 2003). Cooperation, defined as a behavior where two or more individuals work together to achieve a common goal (Boesch & Boesch, 1989), is found in many animals. Examples include cooperative hunting in chimpanzees (*Pan troglodytes*: Boesch & Boesch, 1989) and lions (*Panthera leo*: Stander, 1992), cooperative breeding in which nonbreeding group members help raise their young in meerkats (*Suricata suricatta*: Clutton-Brock, 2002) and azure-winged magpie (*Cyanopica cyanus*: Valencia et al., 2006), and coalition formation in which males work together to monopolize access to breeding females in lions (Bygott, Bertram & Hanby, 1979) and chimpanzees (Watts, 1998). However, it has been discussed in the literature whether such cooperation is based on the understanding of the partners’ role and intentions (Tomasello et al., 2005). This question requires investigating the cognitive abilities of these animals in the experimental studies.

The degree of cooperation and the cognitive abilities required for cooperation can be categorized into four types: recognition of the other’s presence; recognition of the other’s particular action; adjusting actions to those of others in time, space, or both; and adjusting actions as a result of understanding the other’s role (Albiach-Serrano, 2015). To examine the degree to which animals understand a cooperative task, experimental studies have been conducted using a task where two individuals need to simultaneously pull a rope or handles or push buttons (Crawford, 1937; Hirata & Fuwa, 2007; Jaakkola et al., 2018). Previous studies have suggested that the necessity of a partner to solve the cooperative task was understood by chimpanzees (Chalmeau, 1994; Hirata & Fuwa, 2007), orangutans (*Pongo pygmaeus*: Chalmeau et al., 1997), capuchin monkeys (*Cebus apella*: Mendres & De Waal, 2000), Asian elephants (*Elephas maximus*: Plotnik et al., 2011), wolves (*Canis lupus*: Marshall-Pescini et al., 2017), dogs (*Canis familiaris*: Ostojić & Clayton, 2014), and spotted hyenas (*Crocuta crocuta*: Drea & Carter, 2009), because they were more likely to perform the task in the presence of a partner, glance at the partner during the task, and wait for the arrival of a partner, and/or they were less likely to succeed at the task when the visual cues were blocked. In contrast, rooks (*Corvus frugilegus*: Seed, Clayton & Emery, 2008), ravens (*Corvus corax*: Massen, Ritter & Bugnyar, 2015), African gray parrots (*Psittacus erithacus*: Péron et al., 2011), and otters (*Pteronura brasiliensis*, *Aonyx cinerea*: Schmelz et al., 2017) may not do so.

Social and physical factors can also influence the successful cooperation in the experimental task. As an example of a social factor, partners who are more tolerant of each other are more likely to succeed at a task (Massen, Ritter & Bugnyar, 2015). As an example of a physical factor, the use of a long rope in a rope-pulling task increased the likelihood of two individuals to pull the rope together (Hirata & Fuwa, 2007). In addition, complex tasks may hinder success because the task is more difficult to understand (Albiach-Serrano, 2015). These findings require further investigation with different tasks and pairs, even if conducted in the same species.

Cooperation in dolphins has been reported in the wild. Male Indo-Pacific bottlenose dolphins (*Tursiops aduncus*) are likely to form alliances in a pair or a group of three (Connor, Smolker & Richards, 1992). Alliance members herd estrous females together, and they can cooperate to defend or steal females against other alliances (Connor, Smolker & Richards, 1992). Bottlenose dolphins (*Tursiops truncatus*) and killer whales (*Orcinus orca*) may also engage in cooperative feeding. Bottlenose dolphins can create waves and simultaneously rush toward the shore to catch fish (Duffy-Echevarria, Connor & St. Aubin, 2008; Hoese, 1971). One dolphin may drive a school of fish to be surrounded by other dolphins (Gazda et al., 2005). Killer whales have been observed knocking seals off of pack ice by cooperatively creating waves (Pitman & Durban, 2012). These findings in the wild suggest that dolphins may be model subjects for studies on cooperation.

Studies have demonstrated that bottlenose dolphins are also able to solve cooperative tasks in an experimental setting (Kuczaj, Whinship & Eskelinen, 2015; Jaakkola et al., 2018; but see also King et al., 2016). For example, bottlenose dolphins were able to cooperate to open a container that required them to pull a rope simultaneously in opposite directions (Kuczaj, Whinship & Eskelinen, 2015). Jaakkola et al. (2018) used a button-push task, which was accomplished when two subjects pushed buttons together. In this task, the dolphins were able to work together when one individual was provided access to the apparatus after some delay after the first individual was given access. The follower, who started after the initiator dolphin, was more likely to move faster in the initial test, while the initiator, who started before the other dolphin, was more likely to wait for the partner in the later test, and they synchronized the timing of button-pushing with high accuracy. These findings suggest that bottlenose dolphins can understand their partner’s role in a task. In addition, Jaakkola et al. (2018) also reported that the button-push task did not have perceptible causality, and dolphins required more extensive training at the initial stages. Understanding the physical aspects of the task (the consequence of the actions on the apparatus), which may be promoted by the simple causality of the task, may relate to learning an understanding of cooperation (Albiach-Serrano, 2015). Further study using the simple task was required to investigate whether dolphins show similar coordination with the button-push task.

We used a variation of Hirata’s rope-pulling task (Hirata & Fuwa, 2007), which is frequently used as a standard procedure to study cooperation (e.g., Seed, Clayton & Emery, 2008; Plotnik et al., 2011; Ostojić & Clayton, 2014). In this task, two individuals must pull both ends of a rope threaded through a block simultaneously to receive rewards. In contrast, when one individual pulls the rope, the other end of the rope comes out of the block, the block does not move, and the animals lose the opportunity to receive rewards. Therefore, the rope-pulling task may be causally transparent of the requirements to accomplish the task. We investigated how individual bottlenose dolphins coordinate their behaviors within pairs, using delay conditions in which one individual begins the task after the other.

## Materials and Methods

### Subjects and housing

Three bottlenose dolphins (Lusky, Chiku, and Maru) from Kagoshima City Aquarium, Japan, participated in the study and were paired for our experiments; the two pairs were Lusky–Chiku and Maru–Chiku. Lusky was a juvenile male (3 years old at the start of the study). Chiku and Maru were adult females (estimated as 10 and 18 years old at the start of the study). There was no relation among the dolphins. All three dolphins were trained but were naïve in terms of social cognitive experiments. These three individuals were part of a larger group of nine initial dolphins, though the number of members changed during the study period through events such as birth. The aquarium has a main pool (elliptical, 16 m long ×11 m wide, and 5.4 m deep), a subpool (rectangular, 11.3 m long ×4 m wide, and 4.4 m deep), and a care pool (rectangular, 11.5 m long ×3.6 m wide, and 5.4 m deep). Groups of several dolphins lived apart in each pool, and group members often changed among pools. Dolphins did not usually have free access to all pools.

### Ethics Statement

This study adhered to the Ethical Guidelines for the Conduct of Research Animals by Zoo and Aquariums issued by the World Association of Zoos and Aquariums and the Code of Ethics issued by the Japanese Association of Zoos and Aquariums. The protocol was approved by the Animal Research Committee of Kyoto University, Japan (No. 2016–144).

### Apparatuses and experimental setting

**Figure 1:**
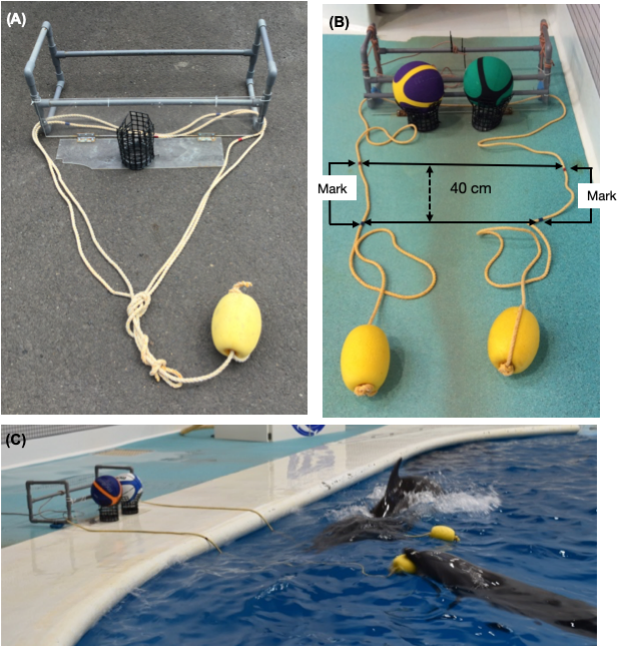
The apparatuses and tasks. (A) The single apparatus, which could be successfully pulled by a single dolphin. (B) The cooperative apparatus, which could be successfully pulled by two dolphins simultaneously. (C) Chiku and Maru performing the no-delay task.

We prepared the trial apparatuses based on a previous study with chimpanzees (Hirata & Fuwa, 2007). We used two types of apparatuses: single (Fig. 1A) and cooperative (Fig. 1B). The single apparatus constituted a box made of PVC pipes (39 × 96 × 35 cm) connected to a board (18 × 71 cm) on which we placed a basketball. A rope was passed through the box, and the ends of the rope were tied together and tied to an elliptical float (13 × 20 cm). This single apparatus with a loop of rope could be successfully pulled by a single dolphin. The cooperative apparatus constituted of the same box and board, on which we placed two basketballs. The rope was passed through the box, and each end of the rope was tied to an elliptical float.

**Figure 2:**
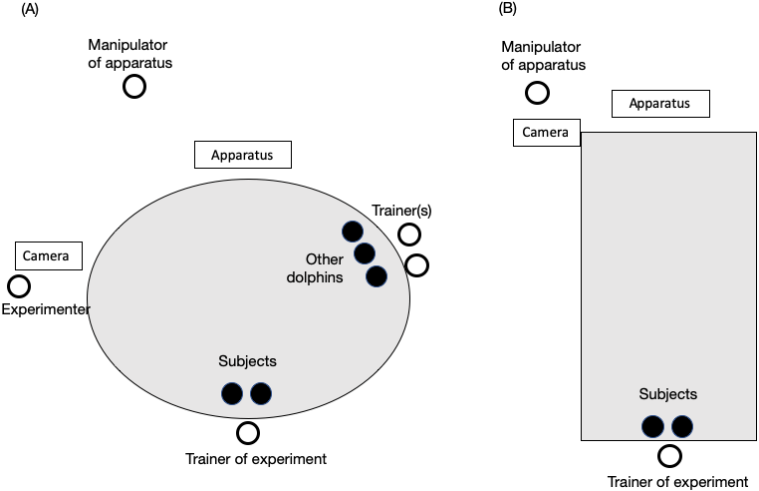
Experimental setting in (A) the main pool and (B) the sub pool. The black circles represent dolphins. White circles represent the trainer or experimenters. The trainer was always one person, and two individuals were always the subjects. The number of other dolphins and trainers paired with them changed throughout the study.

The cooperative apparatus dropped the balls when each dolphin pulled a separate end of the rope together. The length of rope was gradually increased from 40 cm to 200 cm during the no-delay phase for the first pair (i.e., Lusky–Chiku pair); the length remained at 200 cm thereafter. It was changed because one end of the rope would get caught on the side of the pool when a dolphin pulled the other end. Although the length of rope remained at 200 cm, the trial was recorded as a failure when one dolphin pulled the rope over 40 cm, which was judged based on the movement of tape markings. The 40 cm buffer was necessary because one dolphin would hold the rope while waiting for their partner, but the trial was failed if the dolphins did not coordinate their behavior. All experiments were performed in the main pool, excluding the fourth block of experiments for the no-delay phase of the Lusky–Chiku pair to allow for a birth in the main pool; this block of experiments was performed in the subpool (Fig. 2).

### Procedure

#### Preliminary training using the single apparatus

In the preliminary training using the single apparatus, dolphins needed to learn that the ball dropped on the surface by pulling the rope, and they received pieces of fish as a reward when they gave the ball to the trainer. Dolphins swam toward the apparatus at the trainer’s sign that meant “give the trainer the ball,” but we never taught the rope-pulling behavior by active training. Dolphins were able to achieve the task of pulling the rope after several trials. Preliminary training lasted one day for Lusky, two days for Chiku, and nine days for Maru.

#### No-delay phase

Following the preliminary training using a single apparatus, dolphins participated in the no-delay phase using the cooperative apparatus. The trainer presented the gestural sign for both dolphins simultaneously. After the first session, which included ten trials, we reduced the number of trials per session to five because the dolphins were not able to focus on the experiment long enough. Nonexperimental dolphins, including a third subject (Maru), stayed with trainers during the experiments. We finished the no-delay phase when the success rate averaged over 80% across five consecutive sessions. The no-delay phase was conducted during 26 days from June to December 2015 for the Lusky–Chiku pair and 15 days from June to July 2016 for the Maru–Chiku pair.

### Preliminary 3 s delay phase

After achieving the no-delay phase, each pair was exposed to the preliminary 3 s delay phase using the cooperative apparatus to learn that one dolphin (the initiator) should start before the other (the follower). The initiators were always Lusky for the Lusky–Chiku pair and Maru for the Maru–Chiku pair. Chiku was set as the follower in both pairs to decrease the effect of experience in the delay condition, because whether the initiator waited for the partner strongly affected the success of the delay trials in general. One session consisted of 4–6 trials, including both no-delay and 3 s delay procedures presented randomly. Only one session was carried out per day. We finished the preliminary 3 s delay phase when the average success rate exceeded 80% across six consecutive trials. This phase was conducted over 7 days between February and April 2016 with the Lusky–Chiku pair and 31 days between July and September 2016 with the Maru–Chiku pair.

#### Delay test

Following the preliminary 3 s delay phase, dolphins participated in delay tests including 3, 5, and 8 s delay intervals. In the delay test session, the order of each delay condition (3, 5, and 8 s) was set randomly from session to session. Delay trials were separated from each other by a no-delay trial to maintain the dolphins’ motivation. Each pair received one test session per day. Each delay condition was tested in 20 sessions (i.e., 20 trials each for the 3, 5, and 8 s delay conditions; 60 trials for the no-delay condition). The delay test was conducted over 20 days between April and May 2016 for the Lusky–Chiku pair and 20 days between September and December 2016 for the Maru–Chiku pair.

#### Recording behavior

We recorded behavior using direct viewing and video recording (JVC GZ-R70, Kanagawa, Japan, and HITACHI DS-F110, Tokyo, Japan). For tasks using the cooperative apparatus, if both dolphins pulled each end of the rope together and obtained the ball, the trial was scored as a success, and the trainer gave the reward to the dolphins. If only one end of the rope was pulled over 40 cm, the trial was scored as a failure, and the experimenters removed the apparatus and did not give the reward to the dolphins. Failures were categorized into three types. In the first type, called “pulling alone,” one dolphin pulled the rope alone over 40 cm before the other dolphin touched the rope. In the second type, both dolphins pulled the respective ends of the rope together, but one released the rope before dislodging the balls, and then the other pulled the rope over the prescribed length (“release of rope”). For the third type, both dolphins pulled the same end of the rope over 40 cm (“pulling the same rope”).

The latency of rope-pulling was defined as the time from when the trainer gave the dolphin the signal that meant “give the trainer the ball” to dolphin started pulling the rope. We considered that rope-pulling began when a dolphin dove into the water with the rope or moved its head to the opposite side of the apparatus. If its partner failed by pulling alone, we did not measure the latency of rope-pulling for the trial. We defined movement time as the time from when the trainer gave the gestural sign until the dolphin touched the rope (not pulling it) or arrived in the area of the rope. The interval of rope-pulling was defined as the time between when the dolphins began to pull and the successful completion of the trials (interval of rope-pulling was not measured for failed trials).

#### Data analyses

All statistical analyzes were performed using R v. 3.5.1 (R Core Team, 2018). We analyzed the success rate of each phase using a binomial test. In the no-delay phase, five or six consecutive sessions constituted a “block,” and each session consisted of a few trials. We investigated whether the failure ratio for one dolphin pulling the rope alone decreased across the blocks using Spearman’s rank correlation test. The failure ratio was calculated as follows: (the number failures by pulling the rope alone)/(the total number of failures). To show how dolphins changed their behavior in the no-delay phase, we investigated whether latency of rope-pulling by Lusky, who arrived at the apparatus faster than Chiku, or movement time of Chiku changed across the sessions using Spearman’s rank correlation test.

In the delay test, to explore how dolphins coordinated their behavior, we performed three analyses using a generalized linear model (GLM: stats package in R) with the gamma distribution and identity-link functions. The model with the lowest Akaike’s information criterion was selected as the best-fitting model. We also used ANOVA (car package in R) to test whether the model explained significantly more variance when an additional parameter was added. First, we explored whether the initiator modified the time until rope-pulling across the experiments. The dependent variable was rope-pulling latency of the initiator. The predictor variables were delay interval (0, 3, 5, or 8 s) and session number. Second, we investigated whether the follower’s swimming speed was influenced by the initiator’s behavior. The dependent variable was movement time of the follower (i.e., Chiku). The latency of the initiator, delay interval (3, 5, or 8 s), and session number were set as the predictor variables. Third, to explore whether dolphins changed rope-pulling time across sessions, we ran a GLM on rope-pulling interval between two individuals, with delay interval and session number as the predictor variables. For the Maru–Chiku pair, session phase (first- or last-half session) was set as the predictor variable instead of session number, because many trials of the first half failed owing to solo pulling by Maru.

## Results

### Success rate of the no-delay phase

**Figure 3:**
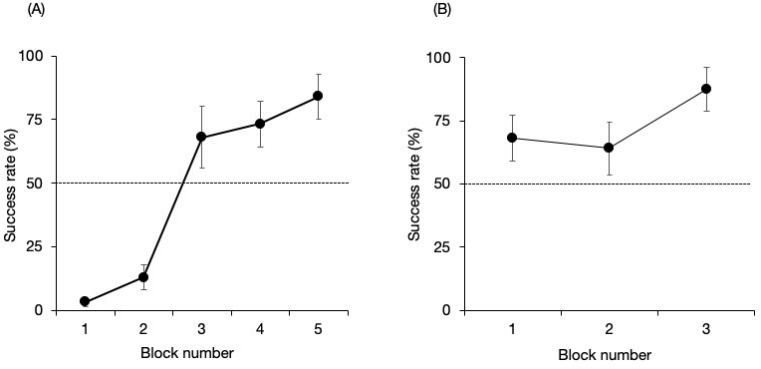
Success rate of the no-delay phase of the (A) Lusky–Chiku and (B) Maru–Chiku pairs. The horizontal line represents the 50% chance level. Error bars show the standard error of the mean.

Figure 3 shows the success rate of the no-delay phase for each pair. There were no significant differences from that expected by chance in the first three blocks but higher than expected by chance in the final two blocks (binomial test one tailed; Block 1, *P* = 1; Block 2, *P* = 1; Block 3, *P* = 0.053; Block 4, *P* = 0.008; Block 5, *P* < 0.001). The Maru–Chiku pair showed better performance during the first block of trials compared to the other pair. They also reached the established criterion within three blocks (binomial test one tailed; Block 1, *P* = 0.053; Block 2, *P* = 0.11; Block 3, *P* < 0.001).

### Failure and behavior changes in the no-delay phase

The “pulling alone” failure by Lusky was the most common (36/69 failure trials), and the “release rope” failure was the second most common (21/69 failure trials). The “pulling alone” failure was most common in the first block and decreased across the blocks (Spearman’s rank correlation test, *r* =  −1, *P* = 0.02). The latency of rope-pulling by Lusky increased with trials (*r* = 0.22, *P* = 0.03), while movement time by Chiku decreased with trials (*r* =  −0.24, *P* = 0.02). In the first and second trials, Maru and Chiku pulled the same side of the rope. From the third trial, they pulled opposite ends of the rope.

### Success of the preliminary 3 s delay phase

In the preliminary 3 s delay phase, the Lusky–Chiku pair had a success rate of 60% from a total of 30 trials. From the first trial, they performed the task successfully. The Maru–Chiku pair had a success rate of 56% from a total of 126 trials. This pair initially achieved success in the fourth trial.

### Success rate of delay tests

**Figure 4:**
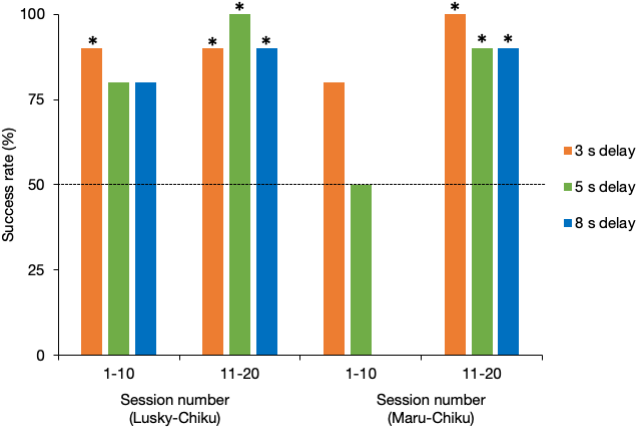
Success rate of the delay test for each pair. The horizontal line represents the 50% chance level. *, *P* < 0.05 (binomial test, one tailed).

Figure 4 shows the success rate of each delay test for the first and second halves of sessions for each pair. The success rate of the Lusky–Chiku pair in the delay test trials was significantly better than by chance alone (binomial test one tailed: 3 s delay test, *P* < 0.001; 5 s delay test, *P* < 0.001; 8 s delay test, *P* = 0.001). Even when separated into the first and second half of sessions, the success rates were higher or tended to be higher than expected by chance (3 s, first half (FH), *P* = 0.01, last half (LH), *P* = 0.01; 5s, FH, *P* = 0.055, LH, *P* < 0.001; 8s, FH, *P* = 0.055, LH, *P* = 0.01).

Success rate in total of the Maru–Chiku pair was better or tended to be better than that expected by chance (3 s, *P* < 0.001: 5 s, *P* = 0.058). However, there was no difference between the success rate for the 8 s delay test and that expected by chance (*P* = 0.75). Success rates in the first halves of the 5 s and 8 s delay sessions, but not the 3 s delay sessions, were not higher than expected by chance (3 s, *P* = 0.055; 5 s, *P* = 0.62; 8 s, *P* = 1), but these rates improved in the second halves of the sessions (3 s, *P* = 0.002; 5 s, *P* = 0.02; 8 s, *P* = 0.02).

### Factors affecting the latency of rope-pulling by the initiators in the delay tests

**Figure 5:**
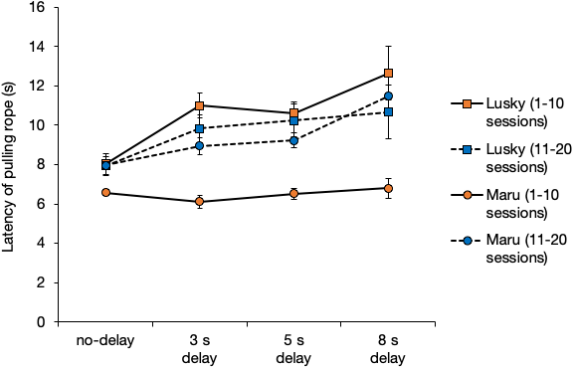
Average latency of rope-pulling for the initiators. Latency of rope-pulling was the time from when the trainer gave dolphins the hand signal. Error bars show the standard error of the mean.

The latency of rope-pulling by the initiator, Lusky, was significantly longer when the delay interval was longer (i.e., from 0 to 8 s) (Fig. 5, GLM: *β* = 0.51, SE = 0.09, *P* < 0.001). The latency of Maru (the initiator) was significantly affected by the delay interval, session, and their interaction (Fig. 5, delay interval, *β* =  − 0.15, SE = 0.060, *P* = 0.01; session number, *β* = 0.12, SE = 0.02, *P* < 0.001; delay interval × session, *β* = 0.03, SE = 0.06, *P* < 0.001). All results of the full model and model selection using GLM are shown in the supplementary material.

### Effect of latency of the initiator on movement time of the follower in the delay tests

**Figure 6:**
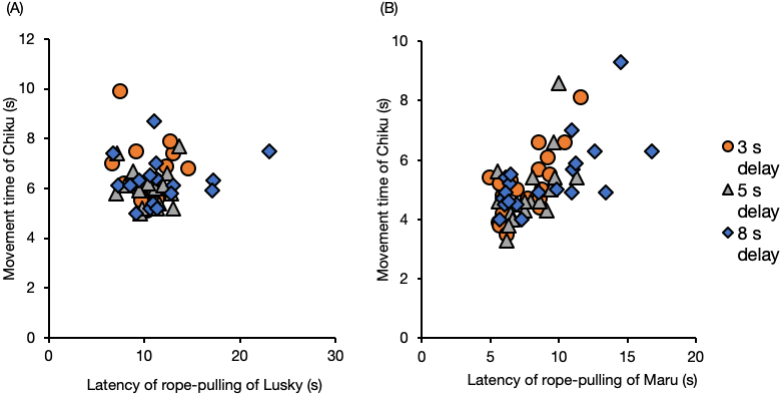
Movement time of the follower (Chiku) for each delay interval with (A) Lusky or (B) Maru as the initiator. Movement time was defined as the time from when the trainer gave her the hand signal until they touched the rope.

The movement time of Chiku (the follower) was not affected by the latency of Lusky, the delay interval, or session number (Fig. 6, Table S10 , GLM). When the initiator was Maru, the movement time of Chiku was significantly prolonged with increased latency of rope–pulling by the initiator (Fig. 6, latency of rope-pulling, *β* = 0.29, SE = 0.05, *P* < 0.001).

### Transition of rope-pulling interval between individuals in the delay test

When the initiator was Lusky, the rope-pulling interval between individuals was not affected by the delay interval or session number (Table S11 , GLM). Lusky pulled the rope but held it in his mouth, after Chiku pulled the rope in 22 of 53 successful trials. In the Maru–Chiku pair, the rope-pulling interval was shorter in the later sessions than in the earlier sessions or when delay interval was shorter (session, *β* =  − 1.31, SE = 0.39, *P* = 0.002; delay interval, *β* = 0.52, SE = 0.11, *P* < 0.001).

## Discussion

We used a modified version of Hirata’s cooperative problem-solving task to show that dolphins successfully cooperated to obtain rewards. Initiators waited for their partner, and the follower swam faster when the initiator did not wait and started to pull the rope. The interval of rope-pulling between individuals decreased across sessions. Our results suggest that dolphins pay attention to their partner’s actions to accomplish cooperative tasks.

The complexity of the physical aspects of a task can hinder a subject’s understanding of the task (Albiach-Serrano, 2015). Although the step of trading the ball for the reward makes our design more complex, we don’t believe this step hindered the subjects’ learning of the task because trading the object for a reward was common for our dolphins. However, whether the rope was moved out of reach for one dolphin, when the other dolphin pulled the rope first, may have affected learning of the task. Coordination under the delayed conditions differed between the pairs. The Maru–Chiku pair employed a strategy in which the follower caught up with the initiator, as observed for bottlenose dolphins in another task (Jaakkola et al., 2018). In contrast, Lusky waited for Chiku to arrive in their sessions. This difference in our pairs may be due to the opportunity for learning a physical mechanism. In a previous study using the rope-pulling task, one side of the rope was moved out of reach of one subject when the other pulled the other side of the rope alone, which may have promoted learning of the physical aspect of the task (Hirata & Fuwa, 2007). It may have been difficult for the dolphins to understand the physical mechanism of our apparatus because the experimenter would remove the apparatus from the dolphins before the rope was moved out of the reach when one subject pulled the rope alone. This was done to prevent accidental ingestion or entanglement in the rope. Lusky failed more frequently than Maru in the initial no-delay phase. He might have “seen” his rope moving from the other dolphin’s rope-pulling when he released the rope before dropping the ball. These experiences may have promoted learning the necessity of behavioral coordination. The Maru–Chiku pair failed more frequently in the early delay condition because Maru pulled the rope alone. Maru, in the early sessions, experienced that her partner was not able to pull the rope after she pulled it alone. In later sessions, Maru waited for the follower, and the accuracy of the Maru–Chiku pair improved. These results support the idea that the experience of impeding the joint action promoted learning the necessity of behavioral coordination.

Dolphins coordinated their swimming speed and latency of rope-pulling with each other in the rope-pulling task, but such coordination did not appear at all times. Previous studies with chimpanzees and orangutans suggest that behavioral coordination appears unidirectional (Chalmeau et al., 1997; Hirata & Fuwa, 2007). The effects of age on coordination were contradictory between studies, so further studies are needed to investigate the effects of social factors such as individual relationship, age, or sex on bidirectional coordination in cooperative tasks.

Bottlenose dolphins solved the rope-pulling task by changing their behavior with a partner. The action of a partner’s rope-pulling and swimming speed may stimulate the other’s rope-pulling and swimming speed. Bottlenose dolphins adjusted their actions in response to the other’s actions, which is one of the types of cooperation (Albiach-Serrano, 2015). It remains to be seen whether coordination in our dolphins was intentional cooperation based on common understanding and sharing intentions. Jaakkola et al. (2018) suggested that bottlenose dolphins understood their partners’ role because the precision of pressing the button simultaneously was high. The improvement in the accuracy of simultaneous rope-pulling across sessions of Maru–Chiku suggests that the bottlenose dolphins in our study understood their partners’ role. However, the interval of action between individuals was longer in our study than in the previous study (Jaakkola et al., 2018). Lusky was more likely to pull the rope after Chiku pulled it. Lusky may have learned to pull the rope when the partner pulled it, or he may be employing a “tricky” strategy, understanding that the task succeeded when he held the rope and the partner pulled it, as was observed in elephants (Plotnik et al., 2011). Differences in the accuracy of synchronous action may have been influenced by the nature of the task, for instance, by the required accuracy or the manner of achieving the task. Further studies are necessary to investigate communication during cooperative tasks and complementary roles to gain more detailed insights into the cooperative behaviors of dolphins.

## Conclusions

In this study, we used a rope-pulling task to investigate how bottlenose dolphins coordinate their behaviors. Each of a pair of dolphins was able to pull a different end of the rope together during the no-delay phase. They adjusted their behaviors to account for the behavior of their partner. In the delay conditions, the initiator waited for the partner, and the follower swam faster to reach the apparatus if the initiator did not wait. The interval of rope-pulling between individuals decreased across sessions. A dolphin was likely to pull the rope after the partner pulled it. Our results suggest that dolphins pay attention to their partner’s actions and coordinate behavior with each other. However, it remains to be elucidated whether coordination in our dolphins represented intentional cooperation. Further studies will investigate the role of communication during the tasks.

##  Supplemental Information

10.7717/peerj.7826/supp-1 Supplemental Information 1 Datasets of each task, and latency of rope-pulling or movement time of dolphins

10.7717/peerj.7826/supp-2 Supplemental Information 2 Successful trial of Lusky-Chiku pair in the delay test

10.7717/peerj.7826/supp-3 Supplemental Information 3 Successful trial of Maru-Chiku pair in second-half sessions of the delay task

10.7717/peerj.7826/supp-4 Supplemental Information 4 Successful trial of Maru-Chiku pair in first-half sessions of the delay test

## Additional Information and Declarations

### Data Availability

The following information was supplied regarding data availability: The raw measurements are available in the Supplemental File.
